# Supplementary material for: Metagenomic data from cerebrospinal fluid permits tracing the origin and spread of Neisseria meningitidis CC4821 in China
Source: Commun Biol. 2022 Aug 18;5:839. doi: 10.1038/s42003-022-03792-0 (PMC9388655; doi:10.1038/s42003-022-03792-0)
Supplement: Supplementary file 2 — Supplementary Information [file 42003_2022_3792_MOESM2_ESM.pdf]

Supplementary Figures

Metagenomic data from cerebrospinal fluid permits tracing the origin and spread of Neisseria meningitidis CC4821 in China

Hongbin Chen [1,#,*], Mei Li [2,#], Shangyu Tu [3], Xiaoyang Zhang [1], Xiaojuan Wang [1], Yawei Zhang [1], Chunjiang Zhao [1], Yinghui Guo [2,*], Hui Wang [1,*]

[1] Department of Clinical Laboratory, Peking University People's Hospital, Beijing, China.

[2] Department of Clinical Laboratory, Children's Hospital of Hebei Province, Shijiazhuang, Hebei, China.

[3] Department of Clinical Medicine, Peking University People's Hospital, Peking University Health Science Center, Beijing, China.

***Corresponding author, email:**

Prof. Hui Wang, whuibj@163.com;

Dr. Hongbin Chen, chenhongbin_pkuph@163.com;

Ms. Yinghui Guo, HeBeiGyh2021@126.com

[#] These authors contributed equally: Hongbin Chen and Mei Li.

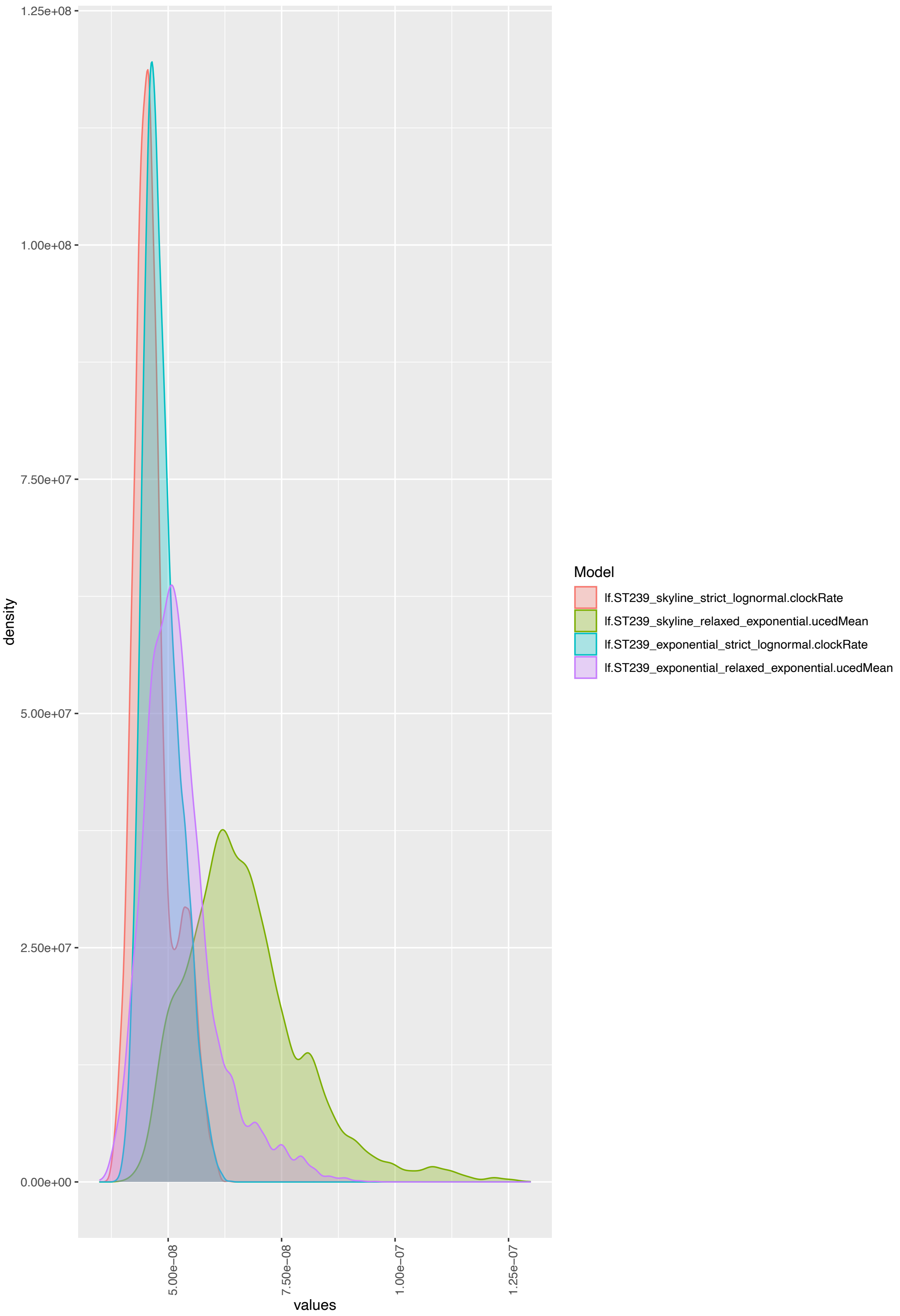

Supplementary Figure 1: **Comparison of evolution rate of different models.**

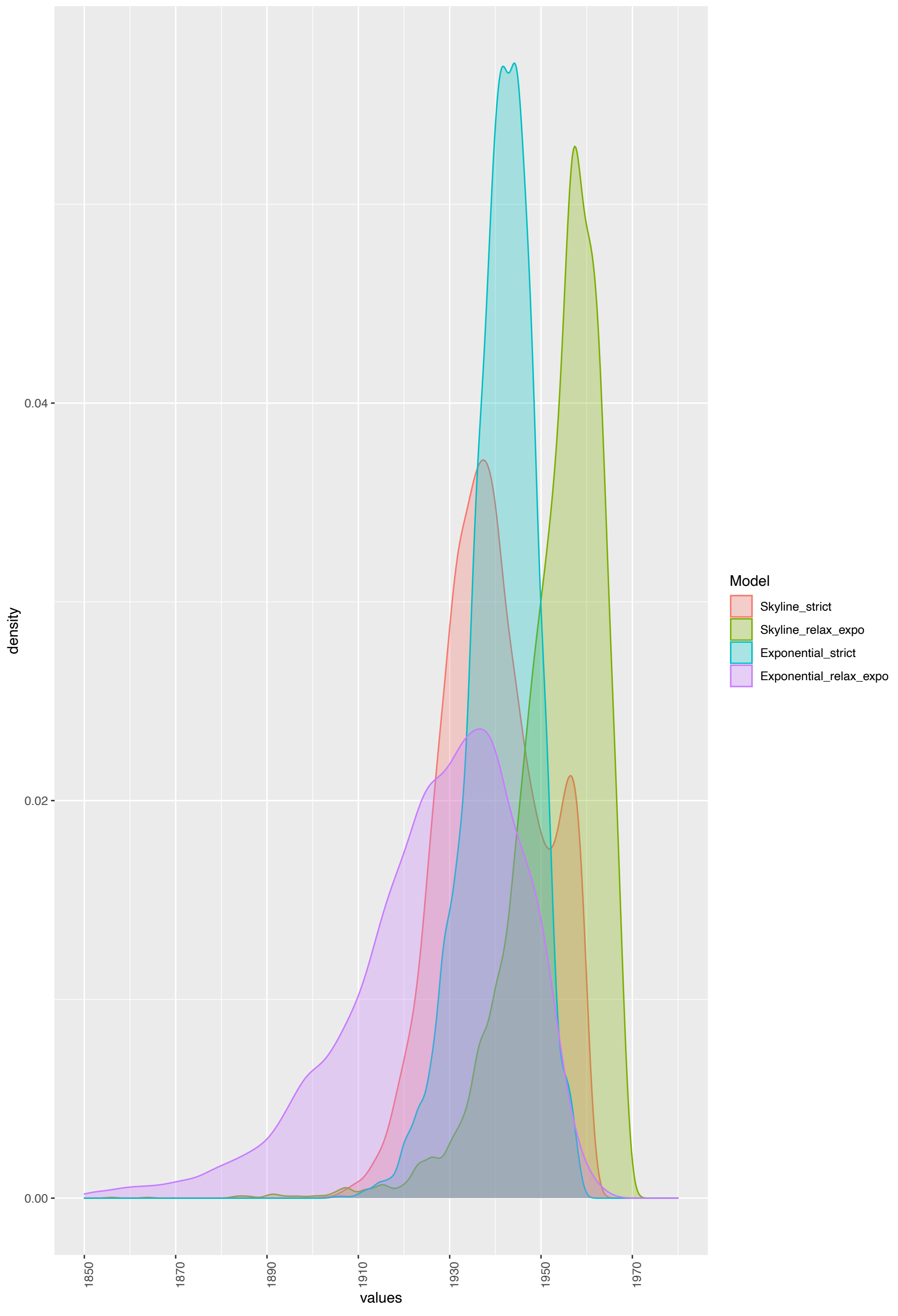

Supplementary Figure 2: **Comparison of the origin time of different models.**

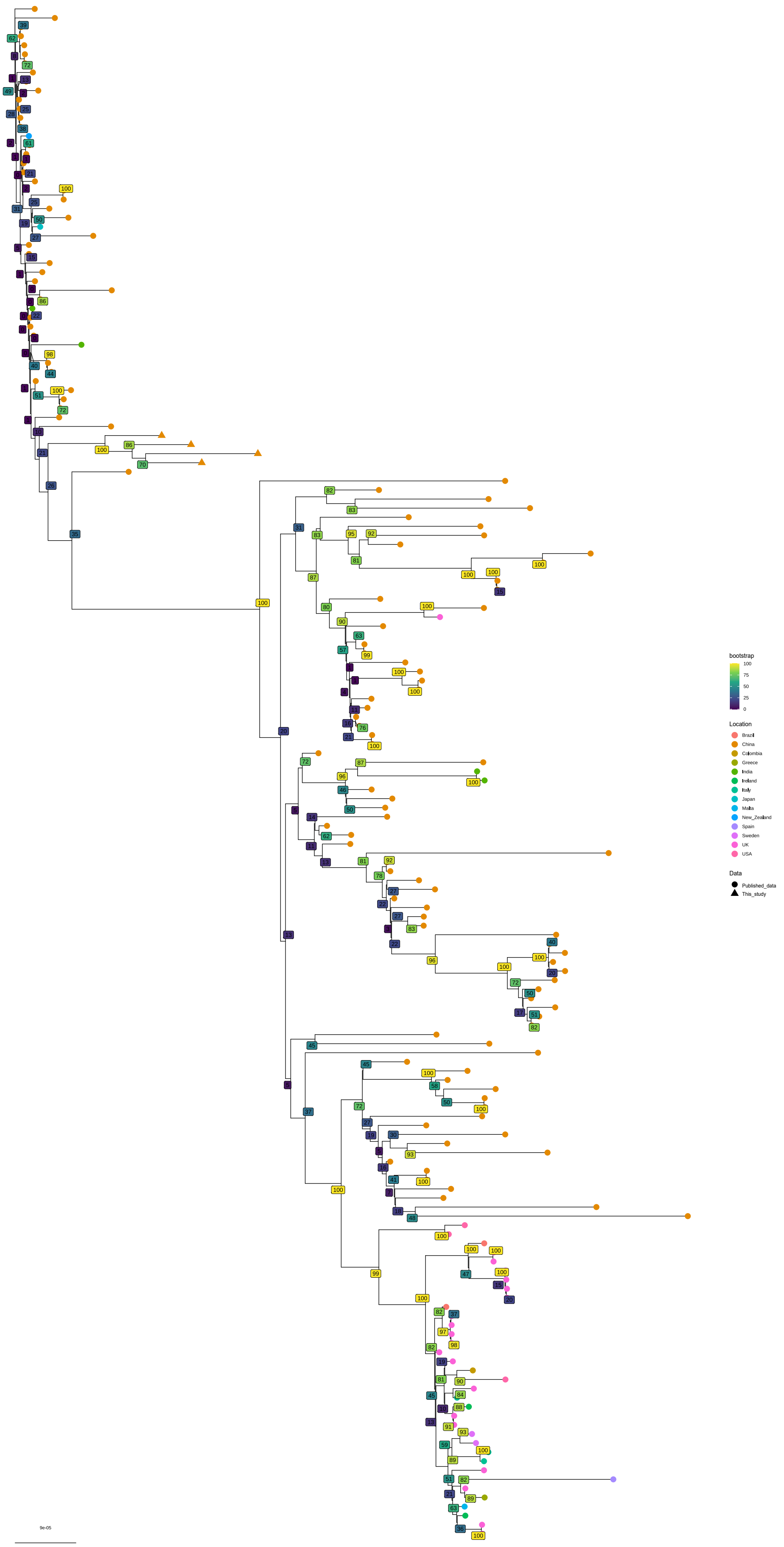
bootstrap
100
75
50
25
0
Location
Brazil
China
Colombia
Greece
India
Ireland
Italy
Japan
Malta
New_Zealand
Spain
Sweden
UK
USA
Data
Published_data
This_study
9e-05

Supplementary Figure 3: **Maximum likelihood phylogeny of CC4821 populations.** The trees were constructed in RAxML v8.2.10 using a GTR model and 1,000 bootstrap replicates. The tips of branches were coloured according to the province from which isolates were sampled. The tree was rooted by *N. meningitidis* MC58. The scale was in units of substitutions per site.

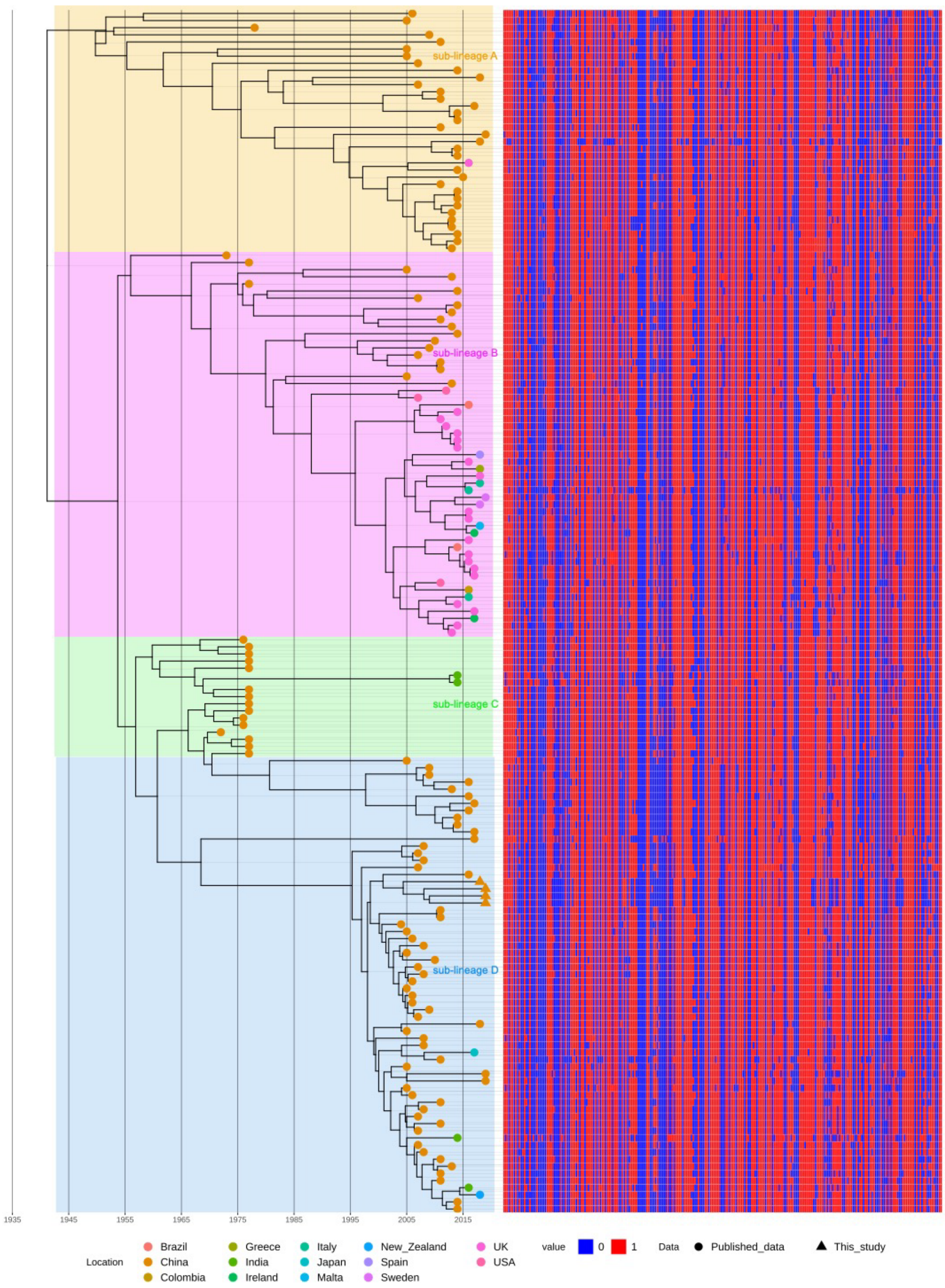

Supplementary Figure 4: **Presence or absence of accessory genes amongst CC4821 isolates.** Accessory genes were identified using Roary v3.11.2. The heatmap of accessory genes was plotted here, with columns corresponding to genes and rows corresponding to individual isolates. Presence of a gene in an isolate was coloured by red, with absence of a gene coloured by green.
