# Supplementary material for: Metagenomic data from cerebrospinal fluid permits tracing the origin and spread of Neisseria meningitidis CC4821 in China
Source: Commun Biol. 2022 Aug 18;5:839. doi: 10.1038/s42003-022-03792-0 (PMC9388655; doi:10.1038/s42003-022-03792-0)
Supplement: Supplementary file 3 — Description of Additional Supplementary Files [file 42003_2022_3792_MOESM3_ESM.pdf]

Description of Additional Supplementary Files

File name: Supplementary Data 1
Description: The source data of the 169 *of N. meningitidis* behind the graphs in the paper.
